# Kinematic Analysis of Pianists' Expressive Performances of Romantic Excerpts: Applications for Enhanced Pedagogical Approaches

**DOI:** 10.3389/fpsyg.2018.02725

**Published:** 2019-01-10

**Authors:** Catherine Massie-Laberge, Isabelle Cossette, Marcelo M. Wanderley

**Affiliations:** ^1^IDMIL, CIRMMT, McGill University, Montreal, QC, Canada; ^2^MPBL, CIRMMT, McGill University, Montreal, QC, Canada

**Keywords:** piano performance, body movement, expression, musical structure, motion capture, motion recurrence

## Abstract

Established pedagogical theories for classical piano usually do not consider the essential relationship between the musical structure, whole body movements, and expression. Research focusing on musicians' expression has shown that body movements reflect the performer's understanding of the musical structure. However, most studies to date focus on the performance of a single piece at a time, leaving unanswered the question on how structural parameters of pieces with varied technical difficulties influence pianists' movements. In this study, 10 pianists performed three contrasting Romantic excerpts in terms of technical level and character, while motion data was collected with a passive infrared motion capture system. We observed how pianists modulate their performances for each of the three pieces and measured the absolute difference in percentage of duration and quantity of motion (QoM) between four expressive conditions (normal, deadpan, exaggerated, immobile). We analyzed common patterns within the time-series of position data to investigate whether pianists embody musical structure in similar ways. A survey was filled in by pianists to understand how they conceive the relationship between body movements and musical structure. Results show that the variation in duration between the exaggerated and deadpan conditions was significant in one measure for one of the excerpts, and that tempo was less affected by the QoM used than by the level of expression. By applying PCA on the pianists' position data, we found that the head QoM is an important parameter for communicating different expressions and structural features. Significant variations in head QoM were found in the immobile and deadpan conditions if compared to the normal condition, only in specific regions of the score. Recurrent head movements occurred along with certain structural parameters for two of the excerpts only. Altogether, these results indicate that the analysis of pianists' body movements and expressive intentions should be carried out in relation to the specific musical context, being dependent on the technical level of the pieces and the repertoire. These results, combined with piano teaching methods, may lead to the development of new approaches in instrumental lessons to help students make independent choices regarding body movements and expression.

## 1. Introduction

While it is common knowledge that musicians' body movements contribute to the audience's understanding of the musical score and the performer's expressive interpretation of music (Vines et al., 2003; Dahl and Friberg, 2007; Weiss et al., 2018), the teacher rarely explicitly guides the students to connect their movements to the structural and stylistic features of a piece (Juslin and Persson, 2002; Young et al., 2003; Karlsson and Juslin, 2008). Although previous research has been conducted on musicians' expressive communication, the impact of the structural parameters of technically challenging pieces on pianists' body movements and expressive parameters remains largely unexplored. The majority of piano pedagogical theories are centered on fingering technique and on the position and weight of the hands and forearms (e.g., Kullak, 1893; Levinskaya, 1930; Wheatley-Brown et al., 2014). Piano teaching would benefit from the inclusion of a science-based pedagogical perspective by incorporating the results of recent experimental studies. A kinematic analysis of experienced pianists' body movements and musical timing in relation to the structural elements from various pieces of music would bring invaluable information that may help student performers monitor their body movements to improve their expressive communication abilities while consistently manipulating acoustical and physical parameters. These results can contribute to the design of a coherent pedagogical framework that may impact piano pedagogy.

In the literature on music performance, two types of gestures have received more attention: effective or instrumental gestures, and sound-accompanying or ancillary gestures (Delalande, 1988; Cadoz and Wanderley, 2000; Wanderley, 2002). Effective gestures are responsible for the direct control of the quality of the sound and changes applied to the instrument itself, while ancillary gestures are not necessarily related to sound production and are mainly the result of three factors: ergonomic, structural and interpretative. The latter are responsible for postural adjustments and they help stabilize the performance, anticipate movements, and maintain the tempo (Godøy et al., 2010; Jensenius et al., 2010). They reflect the performer's individual representation of the music, which is affected by psychological and emotional states. To understand better the functions of body motion in relation to sound, researchers have discussed these different types of gestures, occurring on different timescales, as coarticulated actions (Godøy, 2010, 2013; Jensenius et al., 2010). For instance, a scale played on the piano might seem like a series of separate actions, when considering the finger movements only but those movements are connected and perceived as one coherent gesture if we concentrate also on the movements of the hand, arm, and upper body. In other words, the movements of the whole body may have a perceptual impact on expressive parameters. A recent embodied music cognition theory addresses the close relationships between the musician, his/her body movements and musical instrument, stating that the instrument is a natural extension of the musician's body (Nijs et al., 2018). The musician's body is described as an intermediary between the physical environment and one's personal musical experience (Leman, 2008). The whole-body movements may be so ingrained in a pianist's technique that removing or attenuating some of them may be detrimental to the sound result.

In piano pedagogy, arms and hands are often at the heart of learning the instrumental technique. This approach, although motivated by virtuosity achievement, does not integrate other types of body movements, which coexist with the gestures involved in the production of the sound. To investigate how body movements are connected to musical expression and structural parameters, previous studies used different experimental conditions with gradual levels of expression. Davidson (1993) and Davidson (1994) asked violinists and pianists to perform in three conditions denoted as deadpan, projected and exaggerated. The exaggerated condition was defined as a performance where musicians would exaggerate the acoustic parameters, whereas the deadpan condition would refer to a performance with limited expressive content. In the exaggerated condition, musicians' movements were larger than in the projected one. Moreover, Davidson and Correia (2002) found that while the swaying motion that emanates from the hip region may not be easily visible when pianists perform in a deadpan performance, the motion was still present but at a much smaller scale. However, the relationship between the pianists' swaying action and the musical structure was still not clear (Davidson, 2007). A strong relationship was also observed between pianists' facial expression and body movements, which were linked to specific structural elements of the music (Davidson, 2012). In another study where pianists were asked to play an excerpt from the Beethoven Sonata No.4, Op.102/1, in different modes (i.e., personal, sad, allegro, overly expressive, serene), the quantity of motion was not influenced significantly by the performance modes, whereas the velocity of the head motion was (Castellano et al., 2007). Wanderley et al. (2005) observed the movements of clarinetists while they were asked to perform Stravinsky's Three Pieces for Solo Clarinet in a standard, expressive and immobile manner. The immobile performance consisted of playing the piece with as little movement as possible. The results showed that clarinetists had not suppressed completely their movements, which suggests that certain movements are too ingrained in performers' technique and mental representation to be modified or removed totally. Bell motion in clarinet playing has also been associated to the reinforcement of idiomatic acoustic events at phrase boundaries and at places with harmonic tension (Teixeira et al., 2015). Similarly, Thompson and Luck (2012) used the same three conditions as in Wanderley et al. (2005) and added the deadpan condition previously used in Davidson's research, to examine pianists' movements in relation to the musical structure of the Chopin's Prelude in E minor Op. 28, No. 4. The authors showed that the quantity of motion was modified in specific regions such as the ones with articulations, dynamic markings and at the piece's climax, and that the exaggerated condition was performed with larger quantity of motion in sections with these specific characteristics.

The following studies suggest that musicians' movements are often related to the rhythmic and phrasing structure of an excerpt, as well as to its technical difficulty and character. In order to identify the relationships between the rhythmic structure and similarity in upper body movements, pianists played two Chopin Preludes, similar in character, but different in terms of the phrasing structure (MacRitchie et al., 2009, 2013). Pianists performed different phrases, with analogous rhythmical patterns, from Chopin's preludes using similar motion profiles. This suggests that different pianists shape their movements with respect to the phrasing structure and to the rhythm of the piece. However, while pianists' swaying movements were synchronized with the rhythmical patterns in simple piano pieces, it was suggested that this may not be the case for more complex excerpts (Camurri et al., 2003). Indeed, the periodic swaying motion observed in a pianist's head while performing a Scriabin Etude did not synchronize with the two-bar phrasing structure but was rather correlated with the emotional intensity. The difficulty and the structural characteristics of the different pieces may have had an impact on the synchronization of the movements with the rhythm, as well as on the recurrence of movements between performers.

Other studies investigated the impact of movements on auditors' judgment of musical performances and assessed which parts of the body better convey the expressive intention or emotion of the performance. In piano performance, head and upper torso movements provided meaningful information to auditors, who were asked to discriminate between performance conditions, while the hand movements did not (Davidson, 1994). Camurri et al. (2003) analyzed the expressive movements of a pianist performing a Scriabin Etude in normal and exaggerated conditions and identified that the most efficient auditory and visual cues for the pianist to communicate his expressive intentions were key-velocity, inter-onset-intervals (IOIs) and head movement velocity. Similarly, studies conducted on marimba players investigated the extent to which emotional intentions (i.e., happy, sad, angry, fearful) were conveyed through musicians' movements (Dahl and Friberg, 2007; Dahl et al., 2010). By itself, the head movements appeared to provide sufficient information for observers to recognize the emotions conveyed by the performer. Nusseck and Wanderley (2009) analyzed observers' perception of clarinetists' performances of the Brahms Clarinet Sonata Op. 120, No. 1, when clarinetists' movements are modified. For instance, the motion of different body parts in a video recording was frozen while auditors were judging different parameters. It appeared that freezing the motion of the arms or torso in kinematic displays of clarinet performances do not affect observers' perception of fluency, tension and intensity of the performances (Nusseck and Wanderley, 2009). Moreover, the authors showed that, although performers' movements present consistencies, the total amount of movement and the velocity differ for different body parts. For instance, when one player used larger arm motions, another one performed with more body sway. It was also shown that, during technically challenging passages, the movements seemed to be localized to certain body parts and their amplitude were reduced (Wanderley et al., 2005; Nusseck and Wanderley, 2009). It was suggested that this might possibly prevent fatigue and injury, or may facilitate precise execution.

Expressive manipulations and musical individuality of music performances have been linked mainly to temporal variations (Palmer, 1989; Gingras et al., 2011). Gagnon and Peretz (2003) found that fast tempi were related to expressions of excitement and surprise, while slow tempi were associated with calmness, boredom and sadness. Moreover, a covariation of timing and dynamics tends to occur at the beginning (Clarke, 1987) and the end of phrases (Repp, 1996; Palmer, 1997). Because expression was associated with the magnitude of tempo variations, different expressive conditions were used to evaluate performers' rhythmical strategies to convey these expressions. In their 2005 study of clarinetists' movements, Wanderley et al. (2005) found that the immobile condition was performed faster than the standard and exaggerated conditions, suggesting that motion is associated with the rhythmic structure of phrases. Thompson and Luck (2012) revealed that pianists' tempo was also affected when performing a Chopin Prelude. They looked at each measure separately and found that the exaggerated performances were played slower on average, whereas the deadpan ones were the fastest compared to the standard performances. These tempo variations occurred during specific moments, such as phrase boundaries, or passages with harmonic tension. Contrary to Wanderley and colleagues' findings, the immobile and standard performances were quite similar in duration and pianists could still use tempo variations to perform in an immobile performance. The fact that the deadpan condition was not used in Wanderley et al. (2005)'s study and that the respective complexity of the excerpts in both experiments was different may explain these different results.

Although previous research has focused on the expressive intentions a performer conveys to an audience, it is not clear yet how the structural parameters of musical excerpts with various technical difficulties are embodied in pianists' physical gestures. The study of different Romantic excerpts with various levels of complexity performed by a group of pianists may yield different results that may eventually clarify how auditors perceive and react to musical gestures and expression. This study seeks to understand better how experienced pianists use body movements and timing in relation to structural parameters of pieces with varied difficulties and contexts. First, we evaluate how pianists modulate their performances in terms of duration and quantity of motion (QoM) when asked to play excerpts from the Romantic period in different performance conditions. Second, we investigate how both the structural characteristics of the pieces and the conditions impact the pianists' body movements. Third, we analyze the recurrent patterns of head movement among all pianists when performing in a normal condition. The aim is to visualize where in the score do pianists tend to move in a similar way to understand whether certain movements are dependent on the musical parameters or the physical constraints brought by the instrument. Finally, we assess whether pianists are aware of the way they use body movements in relation to the musical structure and the various expressive conditions. The goal of this research is not to assess whether pianists express their ideas intentionally or not, but to observe the trends and differences among a group of pianists and how various musical excerpts influence body movements and expression. The survey provided us with additional information as regard pianists' expressive decisions and intentions. We hypothesize that the movements from the extremities of the body, such as the ones from the hand or head, will be more accentuated when exaggerating or limiting the expression and that they will vary according to the excerpt performed. We propose that changes in amplitude of movements will be restrained in more demanding passages, such as chromatic passages, and that tempo will be more affected in the deadpan and exaggerated conditions than in the immobile one.

## 2. Methods

### 2.1. Participants and Musical Tasks

#### 2.1.1. Participants

Ten pianists (average of 29.6 years old, *SD* = 5.8, 6 Female 4 Male) participated in this study. The participants were all graduate or post-graduate students (3 doctoral, 3 master's and 4 bachelor's degrees). All participants signed a consent form approved by the University ethics committee.

#### 2.1.2. Pilot Study

In a pilot study, which sought to evaluate pianists' body movements when performing different excerpts in terms of their structural features and technical levels, eleven pianists performed different Romantic excerpts three times in the following order: normal, deadpan, exaggerated and immobile conditions. Similarly to Davidson (1993), Wanderley (2002), and Thompson and Luck (2012), the deadpan condition was described as playing with a reduced level of expression, whereas the exaggerated one, as playing with an exaggerated level of expression. An immobile performance consisted of playing with only the essential movements to produce a normal performance. The high number of excerpts provided data to evaluate multiple parameters of expression such as rhythm, harmony, phrasing, articulation, timing and sound dynamic. Pianists performed each expressive condition three times for a total of 12 performances per pianist. For each pianist, no significant difference in quantity of motion (QoM) was found between all the performances of the same expressive condition. This pilot study allowed us to select three excerpts that demonstrated diverse and contrasting: (1) difficulties, characters and structural characteristics, and (2) data results.

#### 2.1.3. Choices of Excerpts

The three 30-s Romantic excerpts chosen for the current study are listed below:

1. Medtner Sonata Reminiscenza Op.38 (mes. 253–274)
2. Chopin 4th Ballade (mes. 152–160)
3. Chopin Impromptu (mes. 43–51)

Table 1 shows an analysis, conducted by the authors, of the structural characteristics for each excerpt and summarizes the results obtained for each pianist who performed the three excerpts as part of the pilot study.

**Table 1:** Analysis performed by the authors of each excerpt's structural characteristics and summary of results from previous measurements.

**Medtner Sonata Reminiscenza**	**Chopin 4th Ballade**	**Chopin Impromptu**
**STRUCTURAL CHARACTERISTICS**		
-Very dynamic and changing character -Many ascending movements and long arpeggios -Crescendo dynamic -Many accentuated chords and notes -Varied rhythm -Dominant chords -Chromatic passages -Repetitions and modulations	-Impetuous and constant character -Polyrhythm between the hands (constant ternary rhythm at the left hand vs rhythmically unstable melody at the right hand) -Few moments of rest -Chromatic melody with few 8ve intervals that create tension -Repetitions and modulations	-Peaceful and gentle character -Simple melody -Slow and regular rhythm -Smooth dynamics and articulations -Ornaments -Repetitions and modulations
**RESULTS FROM THE PILOT STUDY**		
-All conditions performed faster than normal -Large variations in QoM in the deadpan and immobile performances as compared to the normal condition -Hand movements in the z-axis vary more than other body parts between expressive conditions -Variations in amplitude of hand movement related to the loud dynamic level and accentuated chords -Similar QoM in the normal and exaggerated performances	-All conditions performed slower than normal -Smallest variations in QoM between the normal, deadpan and immobile conditions -Large variations in QoM between the normal and exaggerated conditions -Large amplitude of head motion observed in the exaggerated condition during the return of the main theme and 8ve interval in the melody -Head movement is periodic and follows the rhythm at the left hand, even in the immobile condition	-Exaggerated and immobile performance performed faster than normal and deadpan conditions -Largest differences in QoM between the conditions -Large amplitude of the head motion in the normal performance in the middle of phrases, and at the beginning of phrases for the exaggerated performance -Deadpan and immobile conditions are performed with almost no variations in amplitude of head movement

For the rest of the article, each excerpt will be referred to as the “Sonata,” the “Ballade” and the “Impromptu.” Each excerpt was performed in the same four expressive conditions as used in the pilot study (normal, deadpan, exaggerated and immobile conditions). The pianists played each excerpt once in each expressive condition (for total of 12 performances per pianist). Participants could choose the tempo they found appropriate to convey the expressive conditions. The order of excerpts was randomized for each participant.

### 2.2. Measurements

**Figure 1:**
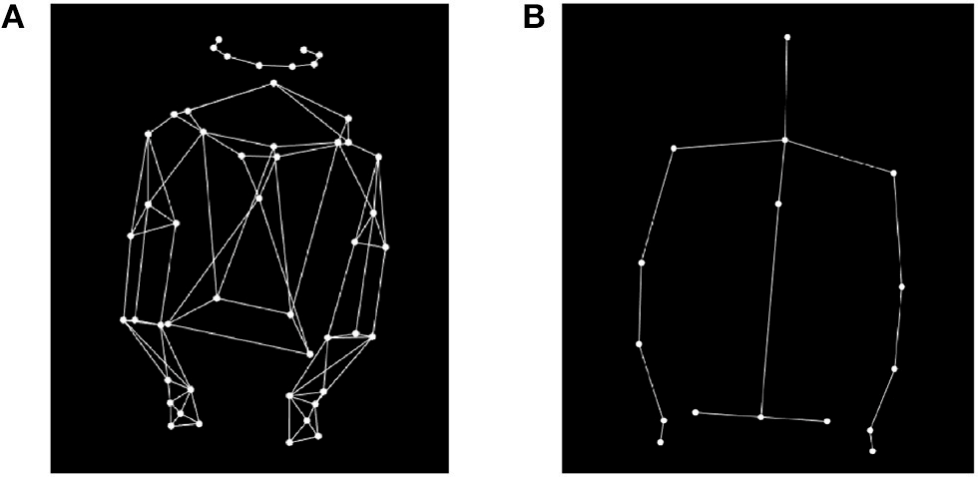
**(A)** Anterior view of the location of markers attached to the pianists' upper body. **(B)** Anterior view of the joint representation of the pianists' upper body.

At the beginning of the experiment, pianists filled in a demographic questionnaire and, at the end of the measurement session, pianists completed a survey to assess how they experienced body movements. Participants were asked questions on their understanding of the structure of the excerpts and how it influenced their musical interpretation. Performances were video recorded with a Sony Wide Angle video camera and audio recorded with a Sennheiser MKH microphone. Motion data were collected, at a rate of 240 frames per second, with a 17-camera Qualisys motion capture system, using 49 passive reflective markers put on the pianists' hands, elbows, shoulders, torso, head, and pelvis. The placement of markers on pianists' upper body and head is shown in Figure 1A. In order to perform the analysis and to extract different kinematic parameters, a set of 16 markers was derived from the marker locations, Figure 1B. The midpoint of a joint was obtained by averaging the location of two or more markers using the MATLAB Motion Capture (MoCap) Toolbox (Burger and Toiviainen, 2013). The beginning of each frame was time-stamped (SMPTE timecode) at 25 Hz, and a Rosendahl Nanosyncs HD word clock, sampled at 48 kHz, generated the clock signals for all the digital devices. The Rosendahl Nanosyncs was connected to the video camera, the Qualisys Sync Unit and the Fireface audio interface. The Qualisys Sync Unit converted the SMPTE signals so that it may be recorded by the mocap cameras. The audio recording was slaved to the video signal. The control computer recorded the audio and MIDI data from the MIDI keyboard with Reaper software and was connected to the same network as the Qualisys computer, which triggered the recordings of both Qualisys Track Manager (QTM) and the audio and MIDI from the keyboard using the OSC protocol.

## 3. Data Analysis

As discussed earlier, previous studies have shown that acoustical and kinematic parameters are important indicators of expression in piano performance. The term *kinematics* is used to describe the spatial details of the movement itself. Kinematics is not concerned with the internal or external forces that cause the movement (Winter, 2009). The present kinematic analysis focuses on the total QoM and the position data in relation to each excerpt's structural parameters. The durations of the performances are also examined with regard to the performance conditions.

### 3.1. Note Extraction and Audio Analysis

To measure the duration of each excerpt in each condition for every pianist, a filter was applied to the absolute value of the audio signal, using the Matlab function movmean, which calculates the moving average across a sliding window. The length of the window used was 200 frames for every participant. Then a sound intensity threshold of 0.001 dB was applied to the signal to mark the beginning and end of each performance. Since pianists could choose the tempo in which to perform each excerpt, the signals also needed to be temporally aligned to the musical structure. Therefore, the exact time of each important gestural event (i.e., notes or beats) was identified and annotated with the audio editor Audacity. The time coordinates of the position data were aligned to their corresponding musical events using a time-warping algorithm (Verron, 2005). All pianists' position data were averaged, time warped and aligned to the score.

### 3.2. Movement Analysis

First, we used principal component analysis (PCA) to determine which body parts vary the most across the performance conditions for each individual pianist. We calculated the cumulative QoM for all the body parts (i.e., head, torso, shoulders, elbows, hands and pelvis) using the MATLAB Motion Capture (Mocap) Toolbox (Burger and Toiviainen, 2013). The QoM of each body part was measured from the joint location data, for each performance condition, in the three axes of the coordinate system. The x-axis represents the motion along the keyboard, the y-axis accounts for the movement toward and away from the keyboard, and the z-axis represents the movement of the body going up and down. This yielded a total of 27 variables for each of the ten pianists playing in the four expressive conditions. We applied PCA on the matrix of kinematic values to reduce the number of relevant features (i.e., body parts and directions of the movements) required to identify which body parts fluctuate the most across conditions for each individual pianist. We identified the first PC and its corresponding feature with the highest coefficient for each pianist. The coefficient is a measure of how each variable contributes to the principal components.

After identifying these body parts, we measured the absolute difference in the total QoM between each condition and the normal condition. We calculated the cumulative distance traveled by the markers to analyze the differences between the expressive conditions. All pianists' cumulative QoM values were averaged together. For each excerpt, the QoM of the normal performances was taken as a reference point (0%) to compare against the values obtained in the other conditions. Then, a series of one-way ANOVAs was conducted for each measure to identify whether there were significant differences between the conditions.

### 3.3. Movement Recurrence

In order to identify the sections of the score in which pianists perform with similar movements and to find the common patterns within the time-series of the position data, we used the instantaneous correlation algorithm developed by Barbosa et al. (2012). The algorithm measures the correlation coefficient between pairs of signals for each frame and generates a bi-dimensional correlation map that reveals the regions of high recurrence between all pairs of signal (i.e., recurrence of movement patterns). The same threshold used in Teixeira et al. (2015) was applied to the map, removing all values below 0.75. We examined the Euclidean norm of the position data together with the correlation map to facilitate the display of the pianists' movement patterns.

## 4. Results

This section reports the results on a) the overall duration of the performance, b) the quantity of motion, and c) the recurrence of movements.

### 4.1. Overall Duration of the Performances

To evaluate how pianists vary the tempi in relation to the levels of expression and the different excerpts, we calculated the duration of every performance (total of 12 per pianist). The lengths of the performances are indicated in Table 2 per pianist and per excerpt. We did not observe any clear pattern between the pianists and conditions in terms of tempi and excerpt lengths: each pianist employed different tempi to perform the excerpts and conditions. Overall, 63% of the deadpan performances and 47% of the immobile performances were performed faster than the normal ones. The exaggerated performances were mostly performed at a slower tempo than the normal ones for all the excerpts (i.e., 8 pianists in the Sonata and in the Ballade, and 7 pianists in the Impromptu). Pianist 2 was the only one to perform all the excerpts slower in the deadpan performance with a percentage difference of 15.33% for the Sonata, 13.52% for the Ballade and 23.77% for the Impromptu, which also corresponds to the largest difference in duration among all pianists.

**Table 2:** Timing of performances of each condition for all pianists.

		**Medtner Sonata Reminiscenza**		**Chopin 4th Ballade**		**Chopin Impromptu**	
	**Performance conditions**	**Time (s)**	**% difference compared to normal**	**Time (s)**	**% difference compared to normal**	**Time (s)**	**% difference compared to normal**
P1	Normal	43.32		25.89		31.97	
	Deadpan	39.21	–9.98	25.08	–3.20	28.57	**–11.23**
	Exaggerated	48.50	**+11.27**	28.98	**+11.24**	34.87	+8.68
	Immobile	41.29	–4.80	23.59	–9.30	30.85	–3.57
P2	Normal	47.88		29.25		31.21	
	Deadpan	55.83	**+15.31**	33.49	**+13.52**	39.64	**+23.77**
	Exaggerated	44.71	–6.86	29.57	+1.09	32.75	+4.81
	Immobile	50.2	+4.73	30.79	+5.13	28.28	–9.88
P3	Normal	40.45		26.98		32.29	
	Deadpan	45.74	**+12.27**	27.10	+0.45	30.77	–4.82
	Exaggerated	40.53	+0.19	23.95	**–11.88**	30.01	**–7.33**
	Immobile	42.44	+4.81	25.86	–4.22	31.33	–3.04
P4	Normal	39.12		24.12		32.60	
	Deadpan	36.48	**–6.99**	21.28	**–12.51**	27.74	**–16.10**
	Exaggerated	41.44	+5.76	26.40	+9.03	34.73	+6.31
	Immobile	39.01	–0.28	24.25	+0.54	33.35	+2.28
P5	Normal	35.59		25.70		39.93	
	Deadpan	38.56	+8.00	25.55	–0.60	38.26	–4.27
	Exaggerated	42.59	**+17.90**	27.05	**+5.12**	42.44	**+6.11**
	Immobile	36.93	+3.69	26.56	+3.28	38.32	–4.12
P6	Normal	37.40		25.85		35.16	
	Deadpan	34.85	**–7.07**	24.27	**–6.32**	31.95	**–9.57**
	Exaggerated	39.55	+5.57	26.38	+2.02	35.46	+0.84
	Immobile	39.25	+4.82	25.80	–0.19	35.95	+2.21
P7	Normal	43.11		39.91		39.27	
	Deadpan	41.06	**–4.87**	41.51	+3.91	38.95	–0.82
	Exaggerated	42.79	–0.75	45.86	**+13.86**	40.53	**+3.15**
	Immobile	43.67	+1.30	42.01	+5.13	39.92	+1.63
P8	Normal	41.20		35.74		36.39	
	Deadpan	43.11	+4.53	37.05	+3.58	36.15	–0.67
	Exaggerated	45.08	+8.98	35.78	+0.10	35.87	**–1.43**
	Immobile	45.34	**+9.55**	39.79	**+10.72**	36.25	–0.38
P9	Normal	47.36		34.74		39.71	
	Deadpan	46.37	**–2.11**	34.36	–1.08	33.76	**–16.21**
	Exaggerated	47.38	+0.04	33.15	**–4.67**	38.20	–3.86
	Immobile	46.65	–1.51	33.90	–2.45	37.49	–5.75
P10	Normal	42.64		41.31		35.47	
	Deadpan	46.07	+7.73	44.12	+6.59	34.56	–2.61
	Exaggerated	48.03	**+11.9**	47.02	**+12.94**	38.47	**+8.12**
	Immobile	43.63	+2.30	43.28	+4.67	33.70	–5.12

*Bold values represented the largest difference as compared to the normal performance*.

**Figure 2:**
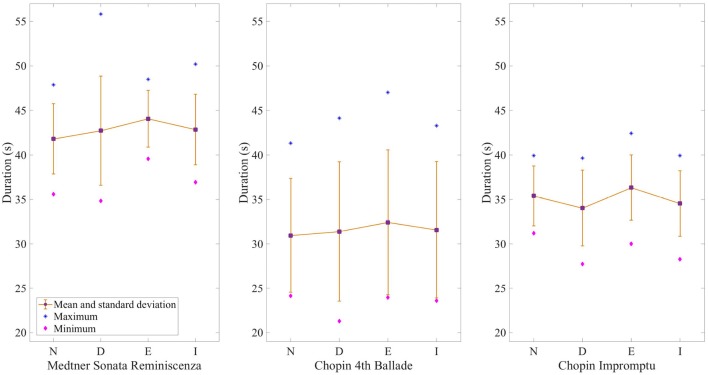
Mean duration of performances for each condition and excerpt. The purple squares show the mean duration and the yellow bars the standard deviation between participants. The blue stars represent the longest performances, while the pink diamonds show the shortest ones. (N, Normal; D, Deadpan; E, Exaggerated; I, Immobile).

**Figure 3:**
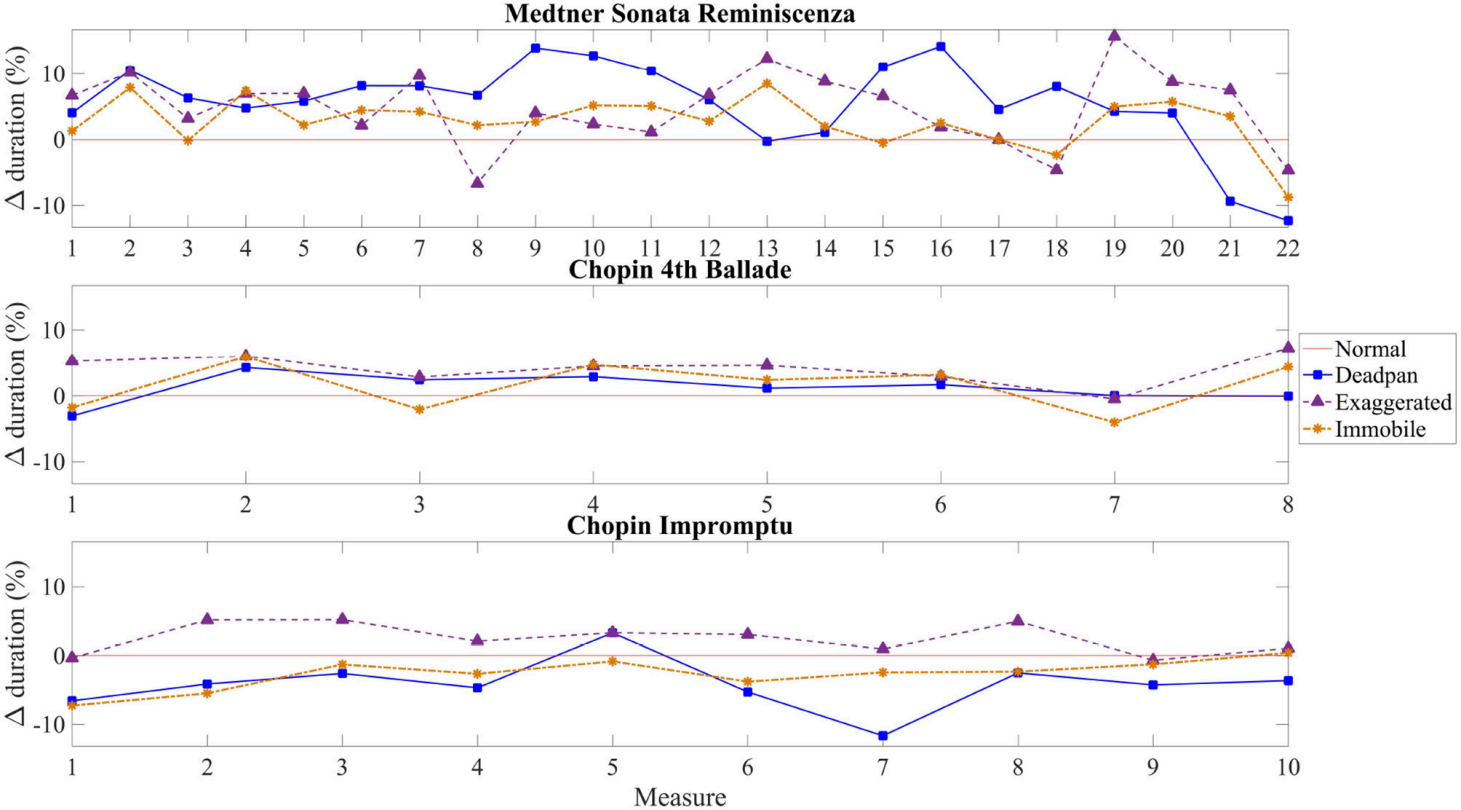
Absolute difference in percentage of duration for each measure per condition. The red line represents the reference point, the normal condition, against which the other conditions are compared.

Figure 2 shows the mean duration of performances and the associated standard deviations between participants and Figure 3 indicates the differences in duration for each measure per condition. The duration of the normal performances, represented by the red line, was taken as a reference (0%) to compare against the values obtained in the other conditions.

#### 4.1.1. Tempo and Musical Excerpts

##### 4.1.1.1. Medtner Sonata Reminiscenza

As demonstrated in Figure 2, the largest average duration for the Sonata occurs in the exaggerated performances (*M* = 44.06, *SD* = 3.19), with a mean percentage difference of 5.24 as compared to the normal performance and the discrepancy among pianists is greater in the deadpan condition (*M* = 42.71, *SD* = 6.14). The smallest deviations in duration from the normal performance occur in the immobile condition with a mean difference of 2.46% slower than the normal condition. The majority of the pianists played the exaggerated (*n* = 8) and immobile (*n* = 7) conditions slower than the normal condition, whereas only half (*n* = 5) of them played the deadpan condition faster. For seven pianists, the smallest variations in duration are observed between the immobile and normal conditions. Although no significant differences were found between the conditions, Figure 3 indicates that the deadpan and exaggerated performances vary more from the normal condition than the immobile performance, but not necessarily at the same places. For instance, while pianists perform the exaggerated condition faster during bars 13 and 14 (fast arpeggio in a crescendo dynamic), these measures are played almost with the same duration in the deadpan condition as in the normal one, whereas the opposite occurs during bars 15–18 (series of accentuated chords).

##### 4.1.1.2. Chopin 4th Ballade

As shown in Figure 2, the durations differ greatly among pianists for all the conditions as exemplified by the high standard deviations, and especially in the exaggerated condition (*M* = 32.41, *SD* = 8.16). The mean duration of the Ballade performed in the four conditions demonstrates smaller differences than for the other excerpts, with a maximum percentage difference of 4.62 in the exaggerated condition (Figure 3). Similarly to the Sonata, most of the pianists performed the immobile (*n* = 6) and exaggerated (*n* = 8) conditions at a slower tempo, whereas five only played the deadpan condition faster. As Figure 3 demonstrates, almost no changes in the measure lengths are perceptible between the expressive conditions.

##### 4.1.1.3. Chopin Impromptu

Contrary to the other two excerpts, pianists tend to perform the immobile condition faster (*n* = 7) in the Impromptu. Most pianists (*n* = 9) performed the deadpan condition faster than the normal condition, with a percentage difference of 4.25. The duration of performances varies almost equally between pianists and conditions, but slightly more in the deadpan performance (*M* = 34.04, *SD* = 4.25). Statistical variations were found for bar 7 only in the Impromptu [*F*_(3, 36)_ = 3.3, *p* < 0.05] as indicated with a Tukey's Honest Significant Test (HSD) (Figure 3). The discrepancy in duration which occurs between the deadpan and exaggerated performances may be explained by the ornaments and rubato during that passage.

### 4.2. Head Quantity of Motion

PCA was used in order to verify which body parts were the most altered when pianists perform in various expressive conditions. Table 3 indicates the first PC and its corresponding component feature with the highest coefficient for all pianists and each excerpt, as well as their respective level of variance across the expressive conditions. The percent variability explained by the first PC provides a sufficiently complex profile to differentiate between the expressive conditions, with a minimum percentage of variance of 85.18 for pianist 10. For the three excerpts, the main component feature that varies the most in terms of QoM across the conditions is the head, and more specifically in the y-axis, that is toward and away from the piano. However, for pianist 2, the right hand is the body part that shows more variations in movement amplitude, in the z-axis (up and down) during the performances of the Sonata, while pianist 10 moves the left elbow with more variations in the x-axis (along the keyboard) during the Impromptu. Moreover, the amplitude of the head in the x-axis differs more for three pianists in the Sonata, for five in the Ballade and for four in the Impromptu. As the PCA revealed that, in general, pianists modulate the amplitude of the head movement when performing various expressive conditions, we decided to analyze more carefully these head movements as relate to the structural characteristics of each excerpt.

**Table 3:** First PC's component feature and level of variance (in %) across all expressive conditions and excerpts for all pianists.

	**Medtner Sonata Reminiscenza**		**Chopin 4th Ballade**		**Chopin Impromptu**	
**Pianists**	**PC1 component**	**Variance (%)**	**PC1 component**	**Variance (%)**	**PC1 component**	**Variance (%)**
P1	Head x-axis	95.89	Head x-axis	95.69	Head x-axis	94.82
P2	Rhand z-axis	94.62	Head y-axis	84.45	Head y-axis	89.41
P3	Head y-axis	95.95	Head y-axis	96.12	Head x-axis	94.56
P4	Head y-axis	96.79	Head x-axis	93.63	Head y-axis	91.66
P5	Head y-axis	95.94	Head y-axis	94.78	Head y-axis	98.01
P6	Head y-axis	98.88	Head y-axis	98.44	Head y-axis	98.48
P7	Head x-axis	91.18	Head x-axis	96.66	Head y-axis	98.66
P8	Head y-axis	89.88	Head x-axis	93.03	Head x-axis	96.95
P9	Head y-axis	97.53	Head y-axis	98.17	Head y-axis	99.00
P10	Head x-axis	85.18	Head x-axis	94.72	Lelbow x-axis	96.50

#### 4.2.1. Head QoM and Musical Excerpts

**Figure 4:**
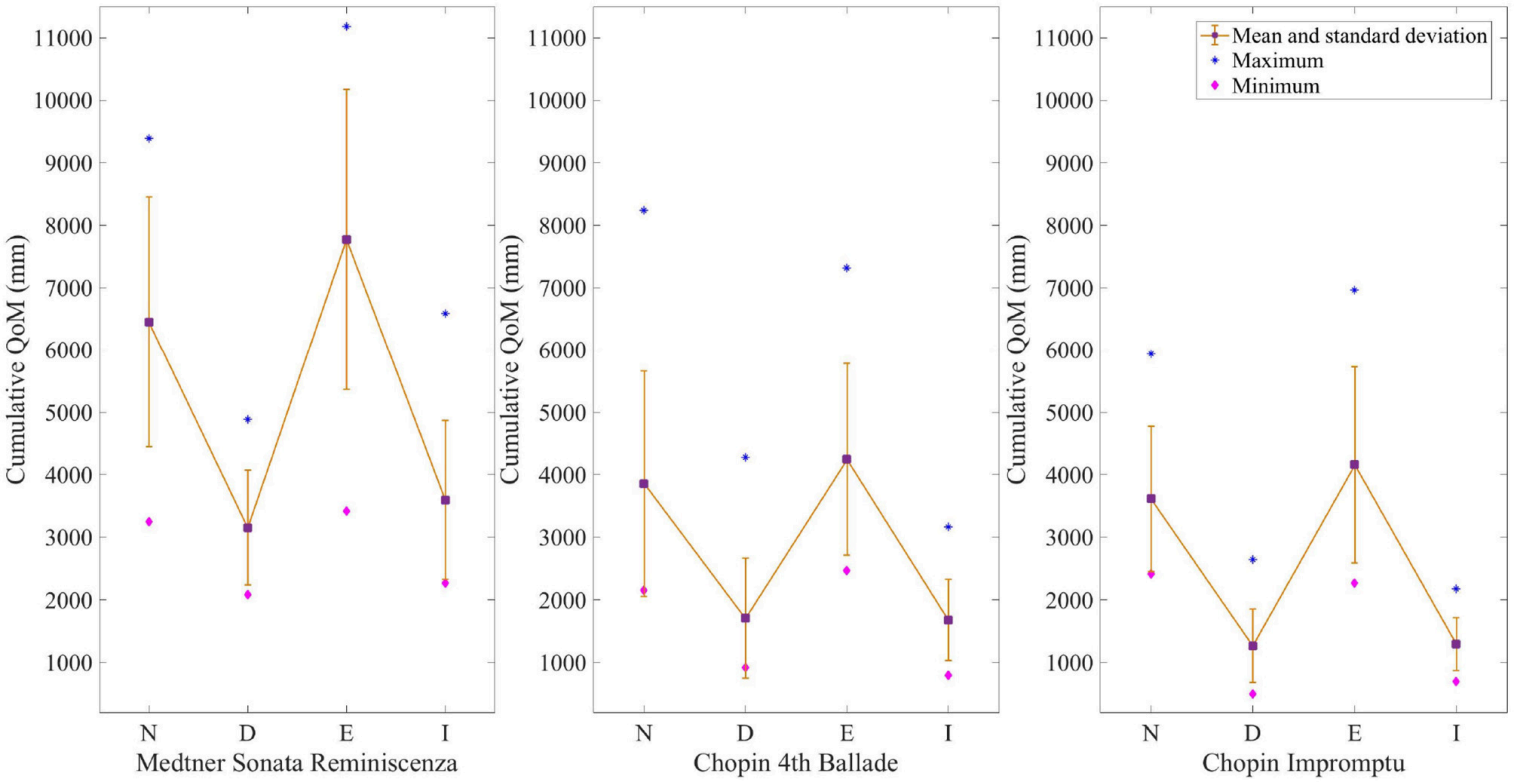
Mean cumulative head QoM for each condition and excerpt. The purple squares show the mean QoM and the yellow bars the standard deviation between participants. The blue stars represent the largest values, while the pink diamonds show the smallest ones. (N, Normal; D, Deadpan; E, Exaggerated; I, Immobile).

**Figure 5:**
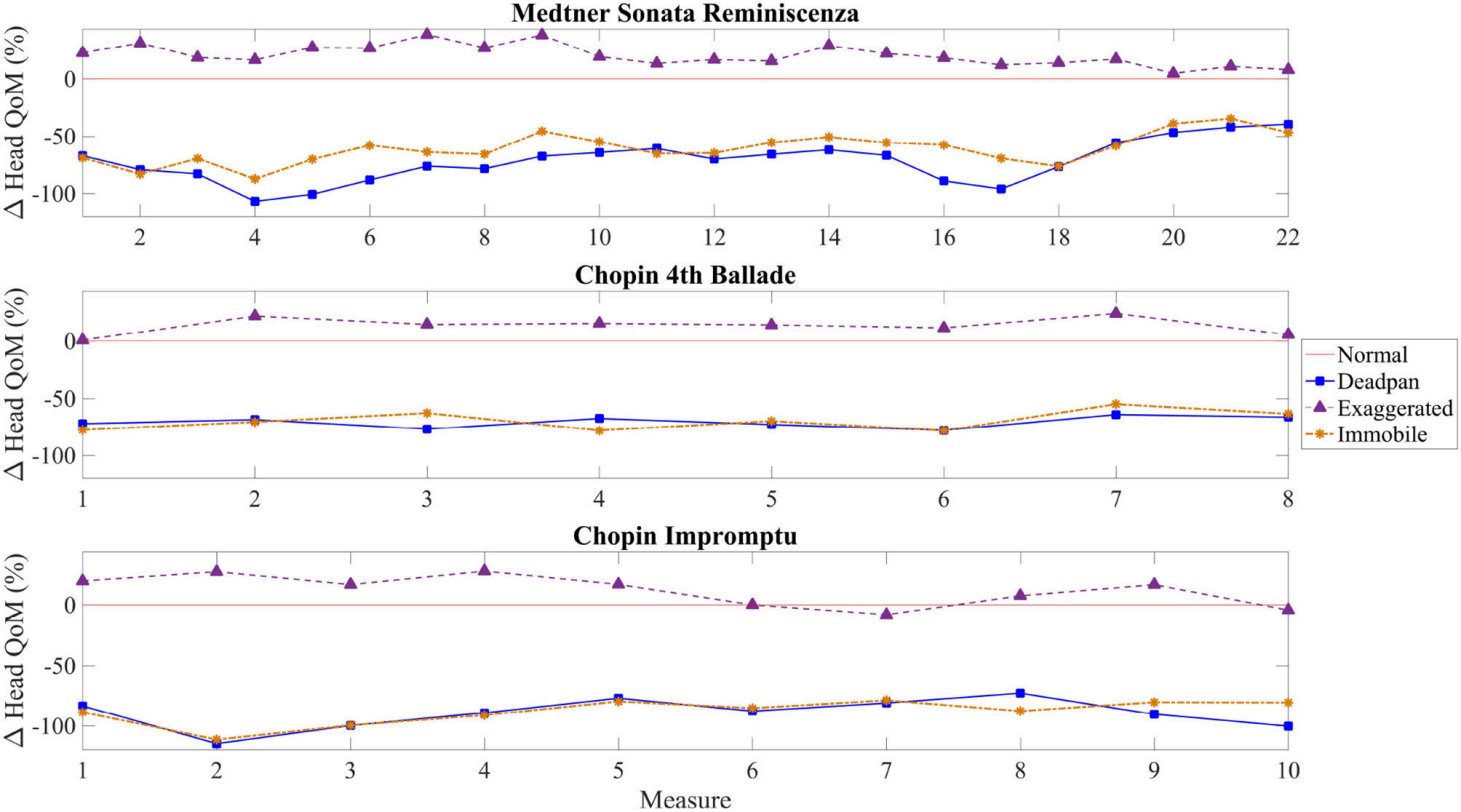
Absolute difference in percentage of head QoM for each measure per condition. The red line represents the reference point, the normal condition, against which the other conditions are compared.

**Figure 6:**
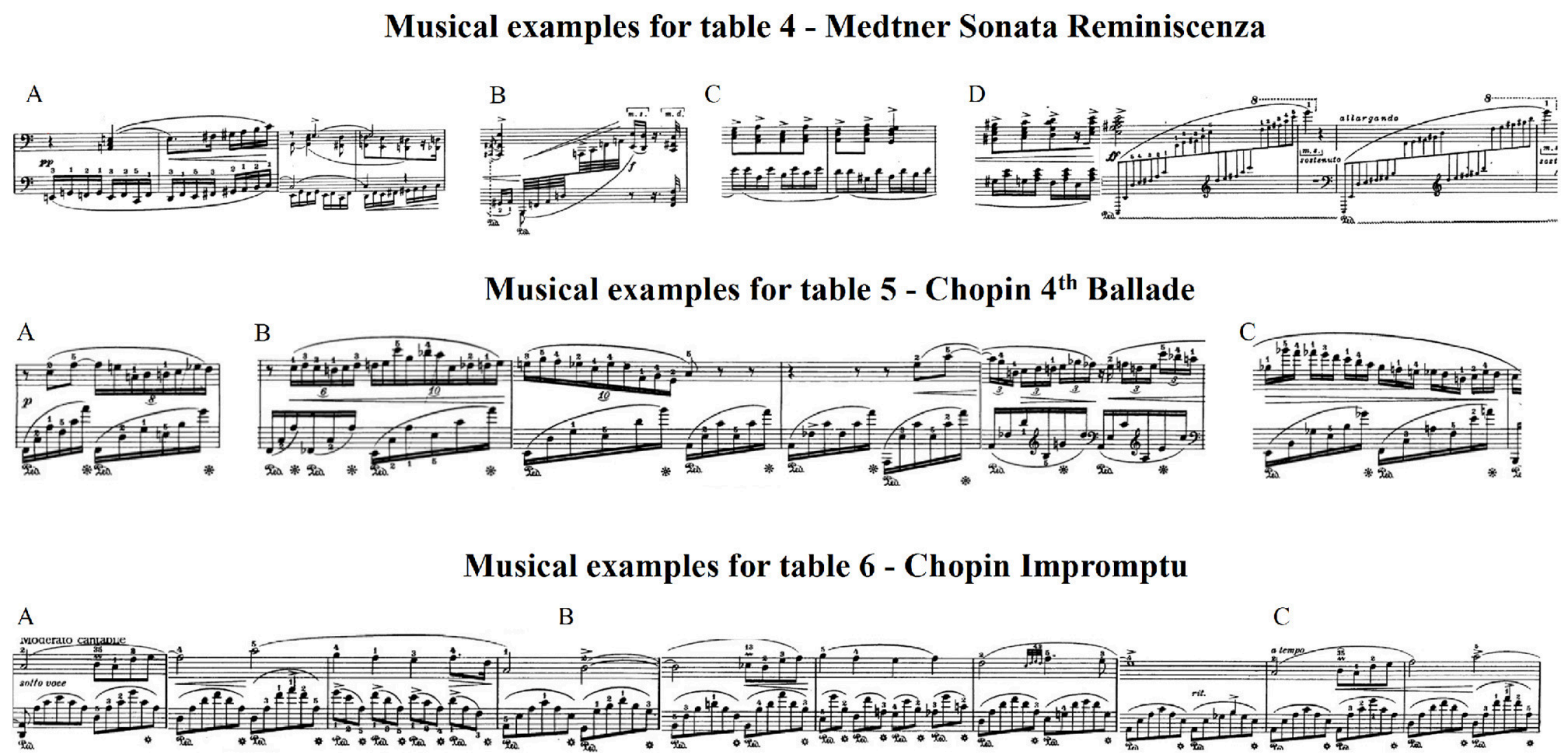
Musical examples for Tables 4–6 corresponding to each excerpt and each region that are significantly different between the expressive conditions.

Figure 4 illustrates the mean QoM and standard deviation for each condition and excerpt. Figure 5 shows the absolute difference of QoM for each measure between the expressive conditions and the normal one. To identify the regions in the score where the amplitude of the head movement differs significantly between the normal and the other expressive conditions, we conducted a series of one-way ANOVAs on the head position data for each excerpt and each measure. A Tukey's Honest Significant Test (HSD) showed which of the expressive conditions differed significantly. The results of the one-way ANOVAs are shown in Tables 4–**6** and the corresponding regions where statistical differences between the conditions occur are displayed in Figure 6. For all excerpts, there was no significant difference between the normal and exaggerated conditions, and between the deadpan and immobile conditions.

**Table 4:** Medtner Sonata Reminiscenza - Results from the one-way ANOVA performed on the cumulative distance traveled by the head marker for the regions presenting significant differences between the normal condition and the other expressive conditions.

			**Tukey's HSD comparisons**	
		***F*_**(3, 36)**_**	***p***	**Conditions**
Region A	Bar 1	11.6	0.009	Normal-Deadpan
			0.01	Normal-Immobile
	Bar 2	11.9	0.05	Normal-Deadpan
			0.04	Normal-Immobile
	Bar 3	10.5	0.007	Normal-Deadpan
			0.03	Normal-Immobile
	Bar 4	12.3	0.003	Normal-Deadpan
			0.01	Normal-Immobile
	Bar 5	11.1	0.01	Normal-Deadpan
	Bar 6	8.9	0.01	Normal-Deadpan
	Bar 7	12.8	0.05	Normal-Deadpan
Region B	Bar 13	11.9	0.004	Normal-Deadpan
			0.01	Normal-Immobile
Region C	Bar 16	6.2	0.03	Normal-Deadpan
	Bar 17	9.2	0.005	Normal-Deadpan
Region D	Bar 19	9.4	0.02	Normal-Deadpan
			0.02	Normal-Immobile
	Bar 20	14.7	<0.001	Normal-Deadpan
			0.001	Normal-Immobile
	Bar 21	13.2	0.002	Normal-Deadpan
			0.007	Normal-Immobile

*The last two rows indicate pair-wise comparisons (Tukey-Kramer) significant at p < 0.05*.

##### 4.2.1.1. Medtner Sonata Reminiscenza

During performances of the Sonata, pianists used on average 20.61% more QoM in the exaggerated condition than in the normal one, significantly higher than for the other two excerpts. Figure 4 shows that the largest discrepancies in head QoM between pianists occur in the exaggerated condition (*M* = 7774.23, *SD* = 2402.15). Differences in mean cumulative QoM between the deadpan and normal, and immobile and normal conditions are larger than between the exaggerated and normal conditions, more specifically between bars 4 and 6 for the deadpan condition, and between bars 16 and 17 (Figure 5). As shown in Table 4, the normal performance varies significantly with the deadpan and immobile conditions in region A (bars 1–4), region B (bar 13) and region D (bars 19–21), and with the deadpan condition only in region A (bars 5–7) and region C (bars 16 and 17). Sections A, B, and D contain ascending chromatic movements in a crescendo dynamic, and the climax of the excerpt is found in section B (Figure 6). Section D starts with a series of fast and accentuated chords, followed by a German sixth chord and a long ascending motion that finishes on a high pitch note at the beginning of measure 21. For all these regions, the head QoM is more reduced in the deadpan performance than in the immobile condition as compared to the normal performance. Pianists did not modulate significantly their movements in the exaggerated condition as compared to the normal condition.

##### 4.2.1.2. Chopin 4th Ballade

As revealed in Figure 4, the smallest variations, across all excerpts, between the exaggerated and normal conditions occur in the Ballade (*M* = 4247.36, *SD* = 1535.17). Moreover, pianists reduce the movement and move the head similarly in the immobile and deadpan conditions (Figure 5). Both conditions mark a clear distinction with the normal and exaggerated conditions. Table 5 shows that significant differences in the amplitude of the head movement between the deadpan and immobile conditions and the normal one occur in three regions. In these three regions, the normal performance differs significantly from both the deadpan and immobile performances, except in bar 3 where it differs significantly with the deadpan condition only. As shown in Figure 6, section A is characterized by the exposition of the theme, section B by a short moment of rest at the right hand before the return of the melody, and section C by a large interval (8ve) in the melody adding tension. The exaggerated condition does not differ significantly from the normal performance.

**Table 5:** Chopin 4th Ballade-Results from the one-way ANOVA performed on the cumulative distance traveled by the head marker for the regions presenting significant differences between the normal condition and the other expressive conditions.

			**Tukey's HSD comparisons**	
		***F*_**(3, 36)**_**	***p***	**Conditions**
Region A	Bar 1	7.1	0.02	Normal-Deadpan
			0.01	Normal-Immobile
Region B	Bar 3	6.2	0.04	Normal-Deadpan
	Bar 4	16.3	0.001	Normal-Deadpan
			<0.001	Normal-Immobile
	Bar 5	9.8	0.008	Normal-Deadpan
			0.01	Normal-Immobile
	Bar 6	6.2	0.05	Normal-Deadpan
			0.03	Normal-Immobile
Region C	Bar 8	26.9	<0.001	Normal-Deadpan
			<0.001	Normal-Immobile

*The last two rows indicate pair-wise comparisons (Tukey-Kramer) significant at p < 0.05*.

##### 4.2.1.3. Chopin Impromptu

As shown in Figure 4, deviations in the head QoM between pianists' performances of the Impromptu are smaller in the normal, deadpan and immobile conditions than for other excerpts (normal: *M* = 3616.89, *SD* = 1163.57; deadpan: *M* = 1265.65, *SD* = 585.02; immobile: *M* = 1291.60, *SD* = 422.96). As shown in Figure 5, the deadpan and immobile conditions require less movement than the normal one, with respectively 89.55 and 88.22% of the movement used during the normal performance. The head QoM in the normal performance differs significantly from both the deadpan and immobile performances for the whole excerpt (Table 6). The excerpt is characterized by a slow modulating melody (region B) and a reiteration of the main theme in the original key (region C) (Figure 6). Surprisingly, for that excerpt, pianists did not modify the head motion significantly between the exaggerated and normal performances.

**Table 6:** Chopin Impromptu-Results from the one-way ANOVA performed on the cumulative distance traveled by the head marker for the regions presenting significant differences between the normal condition and the other expressive conditions.

			**Tukey's HSD comparisons**	
		***F*_**(3, 36)**_**	***p***	**Conditions**
Region A	Bar 1	17.1	0.002	Normal-Deadpan
			0.003	Normal-Immobile
	Bar 2	22.6	<0.001	Normal-Deadpan
			<0.001	Normal-Immobile
	Bar 3	18.8	<0.001	Normal-Deadpan
			<0.001	Normal-Immobile
Region B	Bar 4	14.6	0.004	Normal-Deadpan
			0.005	Normal-Immobile
	Bar 5	9.1	0.04	Normal-Deadpan
			0.02	Normal-Immobile
	Bar 6	12.1	0.001	Normal-Deadpan
			0.001	Normal-Immobile
	Bar 7	8.9	0.004	Normal-Deadpan
			0.002	Normal-Immobile
	Bar 8	10.5	0.01	Normal-Deadpan
			0.002	Normal-Immobile
Region C	Bar 9	17.0	<0.001	Normal-Deadpan
			0.001	Normal-Immobile
	Bar 10	11.8	<0.001	Normal-Deadpan
			0.003	Normal-Immobile

*The last two rows indicate pair-wise comparisons (Tukey-Kramer) significant at p < 0.05*.

### 4.3. Head Movement Recurrence

**Figure 7:**
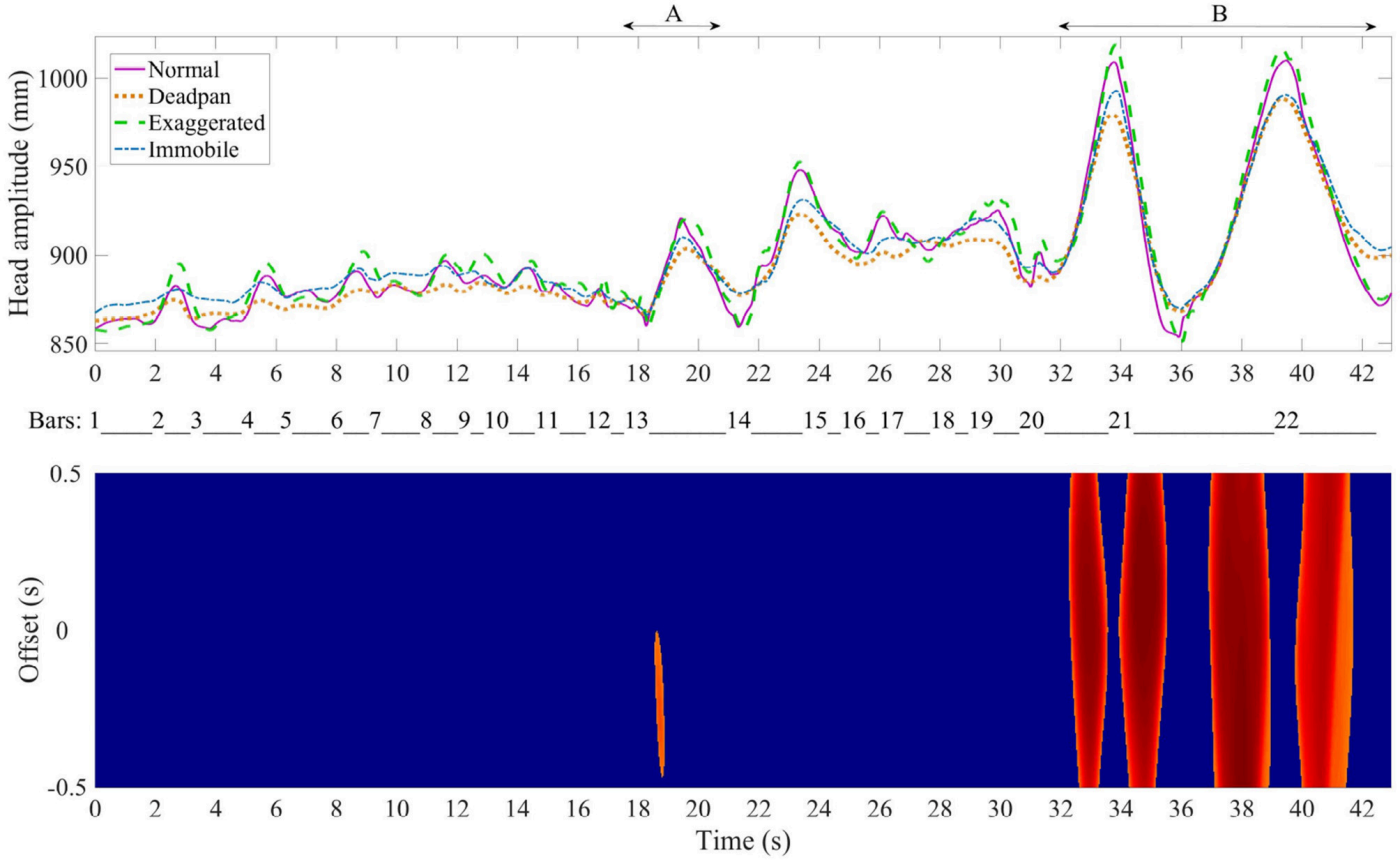
Medtner Sonata Reminiscenza-Top plot: average time-warped amplitude of the head movement in the four expressive conditions. The arrows delimitate the regions of interest. Bottom plot: motion recurrence map indicating the regions with high recurrence (red regions).

**Figure 8:**
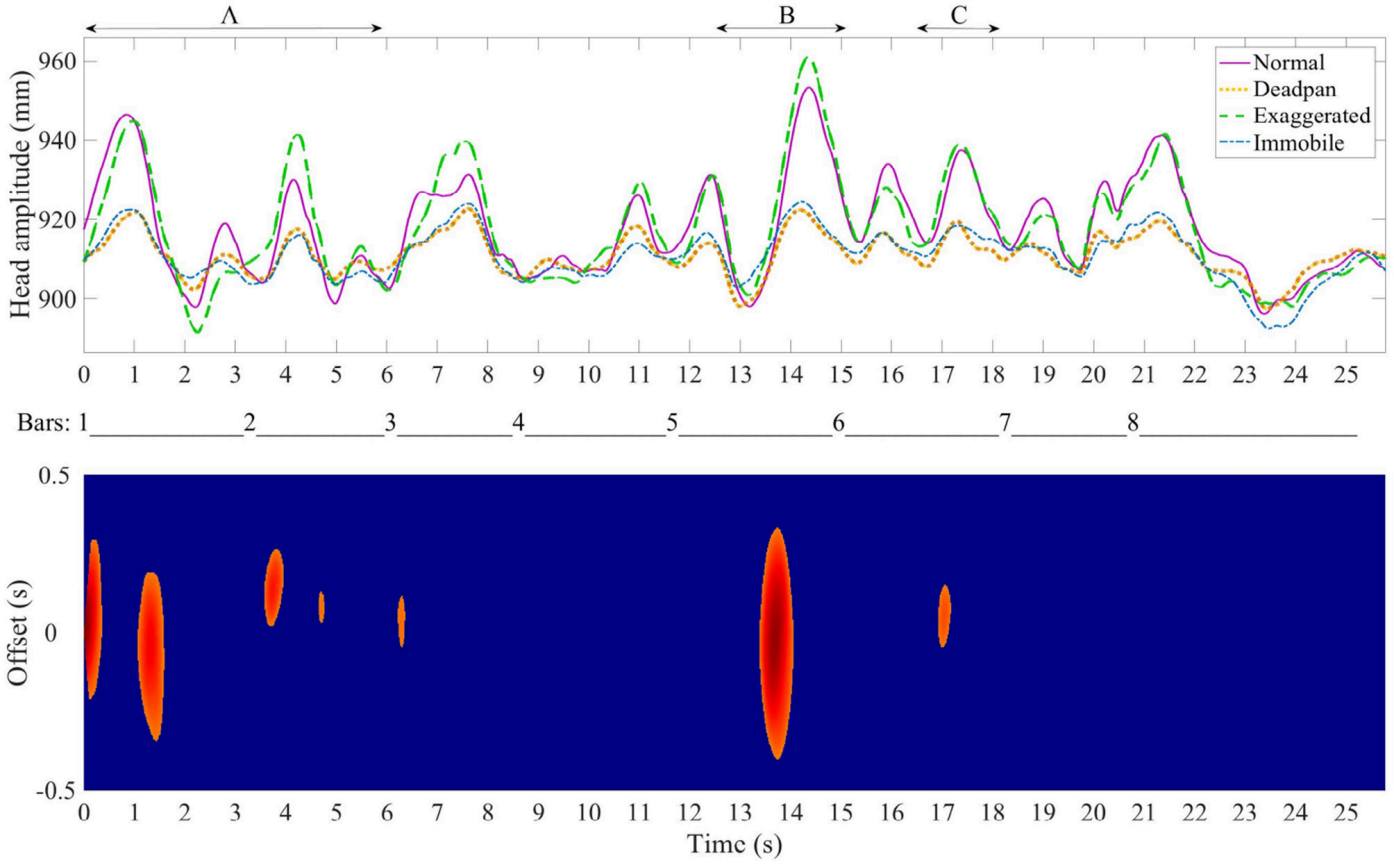
Chopin 4th Ballade-Top plot: average time-warped amplitude of the head movement in the four expressive conditions. The arrows delimitate the regions of interest. Bottom plot: motion recurrence map indicating the regions with high recurrence (red regions).

**Figure 9:**
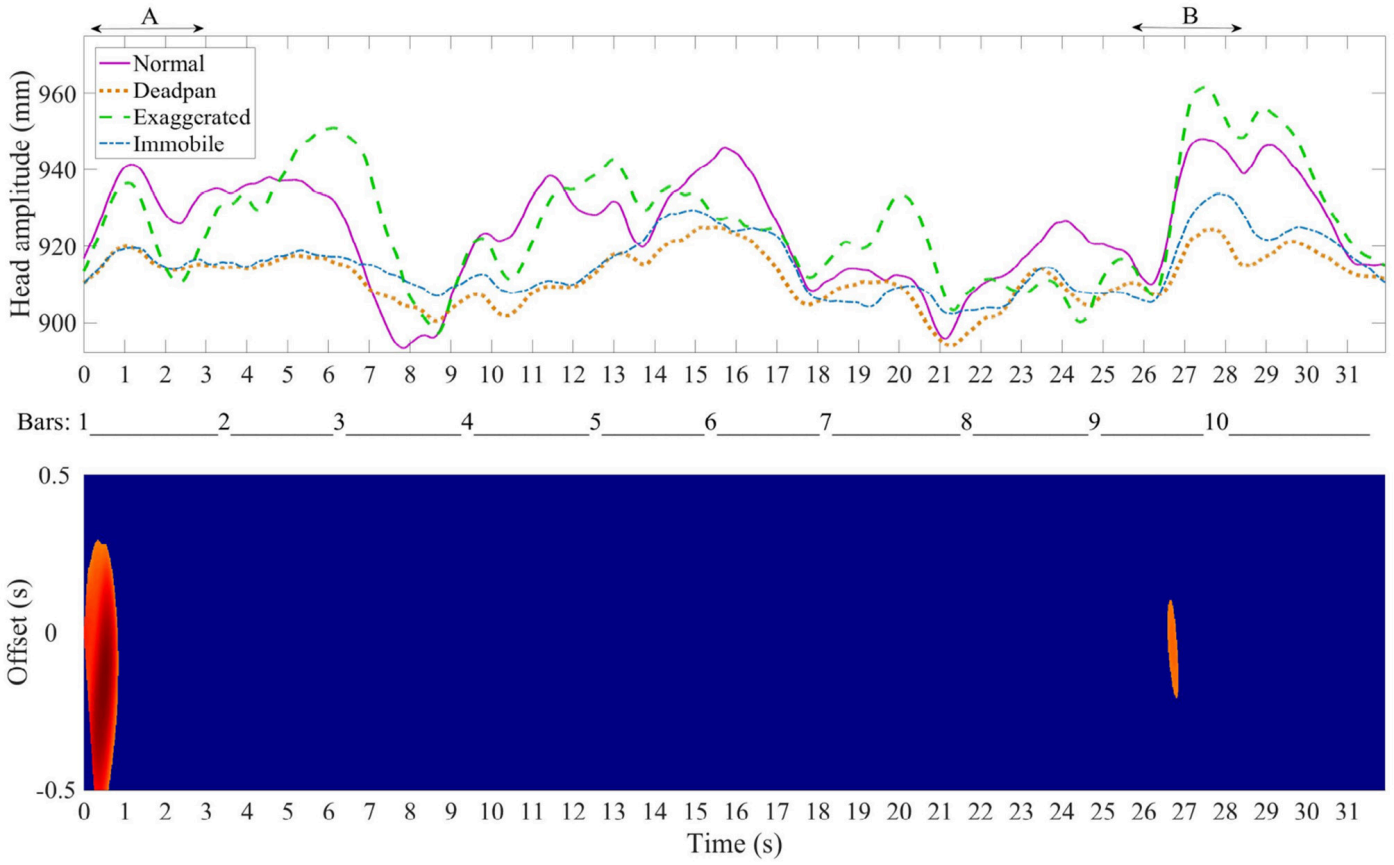
Chopin Impromptu-Top plot: average time-warped amplitude of the head movement in the four expressive conditions. The arrows delimitate the regions of interest. Bottom plot: motion recurrence map indicating the regions with high recurrence (red regions).

To assess whether several pianists embody the musical structure in a similar way, the head position data and the motion recurrence map analysis were used jointly. In the top graphs of Figures 7–**9**, the Euclidean norm of the head position averaged and time-warped are shown in the four different conditions while all the pianists were playing the Sonata, the Ballade and the Impromptu. The bottom graphs show the correlation map which indicates the regions where the pianists used similar head movements. For instance, a large offset in certain regions means that the movement may have been initiated sooner or later depending on the pianist, but that all of the pianists performed with similar movements.

#### 4.3.1. Medtner Sonata Reminiscenza

Figure 7 top graph shows that the changes in head amplitude for the Sonata coincides with rhythmical sections in the excerpts, at bars 13 and 20, which also display high recurrence in the head movement. Bar 13 starts with a large accentuated chord followed by an arpeggio and the last three bars (20–22) are characterized by two arpeggios that span five octaves. Four large offsets of one second are seen at the end of the excerpt, suggesting that pianists initiated the movement with either a delay or a lead of 0.5 s.

#### 4.3.2. Chopin 4th Ballade

The beginning of the Ballade is marked with several regions of recurrent movement patterns, as shown in Figure 8, which coincide with short rests in the melody. Pianists' head movement follows the rhythmic structure at the left hand, a ternary rhythm composed of sixteenth notes grouped in two segments for each measure in the four conditions with amplitude changing on every beat. This effect is more pronounced in the normal and exaggerated conditions than in the deadpan and immobile performances, mainly at the beginning of the excerpt and in the middle of bar 5. Another area where similar head movements are found is in the middle of bar 6, which corresponds to a sixteenth rest.

#### 4.3.3. Chopin Impromptu

As Figure 9 shows, the Impromptu yields large variations in amplitude of the head motion between the conditions, and the deadpan and immobile conditions are performed with a reduced QoM. Only two short regions are performed with recurrent patterns of movements, which is not surprising given the great variations between the conditions. The first region at bar 1 coincides with the beginning of the main theme, which is repeated toward the end at bar 9. The second region is performed similarly among pianists and marks the end of the excerpt on the dominant chord.

### 4.4. Survey

Pianists filled in a survey about their perception of how they move in relation to the musical score. The survey includes open-ended questions related to the strategies pianists employed to convey the different expressive conditions, as well as to the types of movements they used to communicate the musical structure. Pianists' answers to the survey were then used to compare the movement data with pianists' personal assessment of their movements.

- *Question 1*. While performing, do you solicit a specific part of the body? If so, why?

Most of the pianists mentioned that the arms are important for a better control of the fingers and the keys, and to play in a more natural and fluid manner. Using arm weight helps staying connected with the rest of the body and the instrument. The torso and head are generally used to communicate creativity and emotional investment. The hips, although less often mentioned than arms, help project the sound and are used for openness.

- *Question 2*. Are you aware of any specific movements you used to communicate the different expressions?

Most of the pianists stated that during the deadpan performance they decreased the QoM by restricting mainly the motion from the head and arms. The exaggerated condition required them to move with more amplitude, more arm motion and weight, and more hip movement. One pianist perceived that playing in the exaggerated condition created useless tension and imprecision in movements for all the excerpts, but particularly during the Ballade. For the same excerpt, two pianists reported that the immobile condition was easier to perform than the exaggerated condition because for that excerpt, playing with less movement is closer to a natural performance than playing with exaggerated ones. However, for the two other excerpts, pianists found that the immobile manner felt generally unnatural and prevented them from playing fluidly. To perform the immobile condition, they tried to limit the head and torso movements. However, playing with a restricted amount of movements while trying to be natural in the expression helped one pianist identify the regions in the score where excessive efforts were normally made. That pianist mentioned that, while restricting the movements, the focus was put on listening to the performance.

- *Question 3*. For each excerpt, do you think you moved according to the structure of the piece you performed? If so, how?

Pianists said that they used specific movement strategies to convey the respective structural parameters of each excerpt. Overall, pianists mentioned that the movements are mainly connected to the phrase structure, the dynamic shape and the melodic and rhythmic form, and that these parameters influence the amplitude of motion.

#### 4.4.1. Medtner Sonata Reminiscenza

According to the pianists, the Sonata was performed with more hip and torso movements in passages that required playing a series of chords. For them, larger movements from the forearms and elbows were needed for crescendos in this excerpt, while the hips were more implicated before accentuated chords or notes and for attacks.

#### 4.4.2. Chopin 4th Ballade

Three pianists specified that it was difficult to exaggerate the expression in very energetic passages, since these moments already required an investment from the whole body. For instance, for the Ballade, pianists found that the polyrhythm between the hands and the fast displacements of the left hand made it difficult to exaggerate the performance. Many variations in tempo make it difficult to keep a stable rhythmical precision. One pianist mentioned that because of the figurations (i.e., short succession of notes) contained in the excerpt and the many repetitive patterns, special attention on the finger and hand movements was necessary.

#### 4.4.3. Chopin Impromptu

Because of its rhythmic simplicity and uniform writing, most of the pianists found that the Impromptu was the easiest excerpt to perform in different expressive intentions. They also claimed that the expressive variations were mainly done in very melodic parts, which naturally induce larger amplitude of motion in an exaggerated performance. Three pianists specified that fluid and larger arm movements are often used in rubato sections. The moderate tempo of this excerpt therefore gives more flexibility in the movements.

- *Question 4*. Did playing in different expressive conditions affect any particular expressive parameters? If so, which ones?

Pianists revealed that playing in a deadpan manner affected their sense of phrasing and several other expressive parameters, such as tempo and dynamics. Five mentioned that they noticed that their tempo was faster and more stable. They reduced the rubatos, the variations in nuances, as well as the contrasts naturally present between the hands. These same parameters were accentuated in the exaggerated conditions. Four pianists noted that certain regions might have been emphasized, while other passages might have been disrupted by an exaggerated expression because this condition made it difficult to control the sound. Again, most pianists found it difficult to play in the immobile condition, saying that it prevented them from rendering the appropriate expressive result. They mentioned feeling rigid and tense, and as a consequence, they did not perform the dynamic contrasts as well as they would have wanted. On the other hand, one pianist noted that she had the impression that she could play more efficiently while still achieving similar or better sound results.

## 5. Discussion

This paper focused on the kinematic analysis of pianists' body movements in order to understand better how experienced pianists use body movements when performing different Romantic excerpts and when asked to play different performance conditions. We measured the duration and QoM of each performance and identified the regions in the score where pianists use common patterns of head movement.

### 5.1. Duration

We first looked at the variations in duration between the conditions for each excerpt. Although no distinct pattern was found among pianists regarding the overall duration of the performances of each expressive condition and excerpt, we found that the deadpan performances were generally played faster and the exaggerated performances slower as compared to the normal condition. Similarly to the results found in Thompson and Luck (2012), and as *post hoc* pair-wise comparisons showed, the variation in duration between the deadpan and exaggerated conditions was only statistically significant in one measure of the Impromptu. The largest differences between the conditions in tempo were found in the deadpan condition for the Impromptu, whereas the smallest temporal deviations were found between the immobile and normal conditions, more specifically in the Ballade. This suggests that the restricted movement in the immobile condition did not affect the tempo as much as the level of expression in playing. From the questionnaire's results, pianists explained that when they were asked to reduce the level of expression, they used specific strategies, such as keeping a stable rhythm, removing the rubato and reducing the variations at the beginning and ending of phrases, whereas these same parameters were amplified in the exaggerated performances. As opposed to the results found in Wanderley et al. (2005)'s study, the immobile conditions were not necessarily performed faster than the normal ones. This difference may be explained by the fact that the deadpan condition was not used in Wanderley's study. Therefore, the immobile condition, defined as performance with “little movement as possible” where no mention of expression was made could be interpreted differently in their study.

The similarity between the results of the present study, in which pianists performed three Romantic excerpts with contrasting difficulties and those found in Thompson and Luck (2012), where pianists played one Chopin Prelude, indicates that the tempo is generally less affected by the QoM of movement used than by the level of expression regardless of the technical complexity of the piece. It is important to note that not all pianists varied the tempo in the same way to perform the excerpts and the conditions, suggesting that variations are the result of personal interpretative decisions.

### 5.2. Head QoM

Another purpose of the current study was to examine the effect of different pieces with various technical levels on pianist' head QoM and expression. By applying PCA on the pianists' position data, we showed that pianists' head QoM is an important parameter for communicating different expressions and the structural features of various excerpts from the Romantic period, which corroborates results from other studies (i.e., Davidson, 1993; Camurri et al., 2003; Nusseck and Wanderley, 2009; Thompson and Luck, 2012). All pianists performed all the excerpts with less head QoM in the deadpan and immobile conditions as compared to the normal one. Although no specific information as regard the movements was given to the participants for the deadpan condition, pianists considerably reduced their movement, as they did in the immobile condition, which is in agreement with results found in Davidson (1994). This indicates that playing in a deadpan manner may naturally restrict the movements and that movements are intrinsically connected to the expression of pianists. While the duration of the immobile condition was not affected as much as in the other conditions, the QoM, however, was affected in all the excerpts. Interestingly, the pianists used the same amount of head movement during the deadpan and immobile performance of the Impromptu and the Ballade, but not in the Sonata, for which less head QoM was used in the deadpan performance. This result is reinforced by the pianists' answers to the survey which state that remaining static during the immobile performance was facilitated by the fact that the technical challenges of the Sonata already limited the movements during a natural performance.

Davidson (1994) found that pianists performed the exaggerated condition with more amplitude of motion. Although most pianists in this study also performed with more total QoM of the head in the exaggerated condition as compared to the other conditions, it was not the case for each pianist. For instance, the normal condition was performed with more QoM by one pianist in the Impromptu and by two pianists in the Ballade comparatively to the exaggerated condition. Although pianists still varied their movements in the exaggerated condition, the difference with the normal condition was not statistically significant. Since very few indications were given to pianists regarding the execution of the deadpan and the exaggerated conditions, some pianists may have been more reluctant to overly exaggerate the performance than to reduce its expression. As pianists observed in the survey, the technical complexity of the excerpt, such as in the Ballade, may have prevented them from performing with exaggeration without disrupting the flow of the performance.

### 5.3. Musical Structure and Motion Recurrence

Similarly to Camurri et al. (2003), Thompson and Luck (2012), MacRitchie et al. (2013), and Teixeira et al. (2015), we found that pianists' movements and expressive possibilities depend on the underlying structure of the excerpt, but also on its technical level. Variations in amplitude within the time-series of head position data between the conditions and the recurrent patterns in specific regions of the score suggest that certain movements are strongly associated with the structural features of the piece or with the physical constraints of the instrument. The Sonata, which contains more variations in sound dynamics and articulations than the two other excerpts, was performed with more accentuations in the exaggerated condition. Amplitude in head motion between conditions was significantly different in passages with ascending movements and crescendo dynamics. Recurrent head movements were observed when pianists performed wide arpeggios and passages with a chordal texture. Indeed, at certain moments the pianists' movements were dependent on the structure, which created postural constraints and resulted in body weight shifts to the extreme right for all pianists. On the other hand, the technical difficulty of the Ballade, attributed to the complex polyrhythm between the hands, the multiple chromatic passages, and the few moments of rest, prevented pianists from exaggerating the expression and the movements. Although reduced in the deadpan and immobile performances, the movement of the head was synchronized with the periodicity found in the rhythm in all conditions. Pianists moved in similar ways more often during the Ballade, which suggests that the technical level of this excerpt may require specific movements that leave less place for personal interpretative decisions. On the contrary, the Impromptu, characterized by a slow rhythm, smooth dynamics and articulations, gave the pianists the opportunity to emphasize different structural parameters when playing in different conditions. For the Impromptu, large difference in movements between the conditions were observed at the beginning of the melody of the main theme and at the repetition of the same theme, in the deadpan and immobile performances. The correlation map for this excerpt showed that only the beginning of the melodic theme and the end of the excerpt were marked with similar head movements. This means that pianists used distinct expressive movements to perform the conditions and express their personal musical ideas.

### 5.4. Survey

Pianists' answers to the questionnaire gave us important insights regarding the physical and acoustic strategies they can use to convey different levels of expression potentially associated with the musical structure. For most of the pianists, the arms movement and weight are considered as important motion cues to communicate their expressive ideas in a normal performance. Most of them found it difficult to exaggerate the performance in the Ballade, and found that performing in an immobile manner while trying to produce a normal expression was difficult for the Sonata and the Impromptu. For them, it was almost impossible to produce an accurate performance by restricting their movements the way they did.

### 5.5. Conclusion and Further Studies

This research provided new knowledge regarding the types of strategies pianists used to convey expressive intentions and structural parameters through body movements. Although pianists used varied strategies in terms of tempo and QoM to communicate different expressions, we identified similar trends in specific areas of the score. Our results indicated that when ten pianists performed three excerpts from the Romantic repertoire in difference expressive conditions (normal, deadpan, exaggerated and immobile): (a) the duration of performances was less affected by the QoM used than the level of expression regardless of the technical level of the excerpt, (b) the head QoM communicated well different expressions and structural features, and was only significantly different in the immobile and deadpan conditions when compared to the normal condition for all the excerpts, but mainly during the Impromptu, (c) the Sonata allowed more variations in amplitude of the head movements in the exaggerated condition than the two other excerpts due to the variety of elements in the writing, whereas the complex polyrhythm and melody in the Ballade prevented pianists from performing with exaggeration in the movements, and (d) recurrent head movements were found in specific regions of the score for the Sonata and the Ballade only. The results of this kinematic analysis, combined with common piano teaching methods, can benefit the field of piano pedagogy by helping teachers implement and integrate a more systematic approach in instrumental studio lessons in terms of accurate feedback related to movements and musical expression. Learners would be able to compare their movements to those of experienced pianists and become aware of the effect of that movement on the communication of expressive and structural parameters. Providing more systematic feedback in instrumental lessons can help students transfer teachers' explanations to various musical contexts so they may make independent creative choices, and aim to increase their musical communicative abilities.

Further studies investigating the ability of auditors to discriminate between a normal and immobile conditions could help evaluate whether reducing the movements in a performance affects auditors' perception of musical expression. The authors of this article have shown that even a slight modification in movements, such as the amplitude or acceleration of head motion can influence the sound parameters in a way that is noticeable for auditors (Massie-Laberge et al., unpublished manuscript). Additional work is also needed to identify whether there are distinct groups of pianists who tend to perform with similar body movements and whether these groups differ in terms of individual musical formation, influences and pianistic styles. Extensions of this work could also consider the impact of pieces from various musical periods on pianists' movements. Finally, expressive parameters, such as loud sound dynamics, accents, fast rhythms and rich texture, can be heavily dependent on the motion coming from the hip region. Complementary studies may examine the co-variations between the force applied on the piano stool and body movements to understand further the mechanisms involve in the movements, as well as weight compensation strategies used by pianists.

## Data Availability Statement

The raw data supporting the conclusions of this manuscript will be made available by the authors, without undue reservation, to any qualified researcher.

## Ethics Statement

This study was carried out in accordance with the recommendations of the McGill University Policy on the Ethical Conduct of Research Involving Human Participants and the Tri-Council Policy Statement: Ethical Conduct For Research Involving Humans, McGill University Research Ethics Board II (REB-II). All participants gave written informed consent in accordance with the Declaration of Helsinki. The protocol was approved by the McGill University Research Ethics Board II, a unit within the Office of the Vice-Principal (Research&Innovation). REB File number: 101-0815.

## Author Contributions

CM-L, IC, and MW: design of the experiment; CM-L accomplishment of the experiment, data processing and analysis, and writing of report; IC and MW: research supervision and review of the report.

### Conflict of Interest Statement

The authors declare that the research was conducted in the absence of any commercial or financial relationships that could be construed as a potential conflict of interest.
